# Determinants of microbial colonization in the premature gut

**DOI:** 10.1186/s10020-023-00689-4

**Published:** 2023-07-05

**Authors:** Xiaoyu Chen, Yongyan Shi

**Affiliations:** Department of Pediatrics, Shengjing Hospital of China Medical University, Shenyang, 110000 China

**Keywords:** Gut microbiota, Microbial colonization, Dysbiosis, Preterm infant, Human breast milk, Antibiotics, Probiotics, Delivery mode

## Abstract

Abnormal microbial colonization in the gut at an early stage of life affects growth, development, and health, resulting in short- and long-term adverse effects. Microbial colonization patterns of preterm infants differ from those of full-term infants in that preterm babies and their mothers have more complicated prenatal and postnatal medical conditions. Maternal complications, antibiotic exposure, delivery mode, feeding type, and the use of probiotics may significantly shape the gut microbiota of preterm infants at an early stage of life; however, these influences subside with age. Although some factors and processes are difficult to intervene in or avoid, understanding the potential factors and determinants will help in developing timely strategies for a healthy gut microbiota in preterm infants. This review discusses potential determinants of gut microbial colonization in preterm infants and their underlying mechanisms.

## Background

According to the World Health Organization (WHO), preterm infants (PTIs) are those born at < 37 weeks of gestation. Every year, 15 million PTIs are delivered worldwide, which accounts for more than 10% of all deliveries (Harrison and Goldenberg 2016). Prematurity remains the leading cause of neonatal mortality and is associated with an increased risk of deficits in cognitive outcomes and neurodevelopmental disabilities in childhood (Serenius et al. 2013; Cheong et al. 2018; Crump et al. 2019; Zhu et al. 2021; Husby et al. 2023). In the short-term, PTIs are more likely to experience intestinal injury due to their fragile intestinal barrier (Lemme-Dumit et al. 2022; Ma et al. 2022). 90% of necrotizing enterocolitis (NEC) cases occur in PTIs, and the severity of NEC is associated with the degree of prematurity (Kosloske 1994). This life-threatening intestinal disease is a major cause of morbidity and mortality in PTIs, posing a significant threat to global public health (Stoll and Hansen 2003; Battersby et al. 2018; Healy et al. 2022). A higher risk of other intestine-associated diseases is also found in PTIs (Healy et al. 2022; Humberg et al. 2020).

The gut microbiota (GM), which includes trillions of microorganisms inhabiting the digestive system, is complex and dynamic (Brody 2020). Starting from birth, the GM performs important functions in digestion, nutrition, and growth, as well as participating in the maintenance of intestinal epithelial homeostasis, activation and maturation of the immune system, and resistance to pathogens (Gomez et al. 2016; Dominguez-Bello et al. 2019; Henrick et al. 2021; Kalbermatter et al. 2021; Durda-Masny et al. 2022). The microbiota in the premature gut has attracted much attention because of its impact on PTIs, especially intestinal diseases. With the rapid development of metagenomic studies in recent years, the composition and function of the preterm GM has been extensively investigated.

GM colonization starts from, if not earlier than, the initiation of labor. The early period after birth plays a vital role in the establishment of the GM. Patterns of microbial colonization in PTIs differ from those in full-term infants (FTIs) owing to prenatal factors, birth mode, feeding type, and antibiotic use(Aguilar-Lopez et al. 2021). Dysbiosis at the early stage of life is likely to predispose PTIs to NEC and late-onset sepsis (LOS) (Jacob 2016; Warner et al. 2016; Pammi et al. 2017; Stewart et al. 2017). Dysbiosis is also associated with higher risks of childhood obesity, asthma, IgE-associated eczema, autism, and neurodevelopmental impairments (Pammi et al. 2017; Boghossian et al. 2013; Shreiner et al. 2015; Luca and Shoenfeld 2019; Marietta et al. 2019; Musis et al. 2020; Fu et al. 2021; Lee et al. 2021). These findings emphasize the essential role of microbial colonization.

**Fig. 1:**
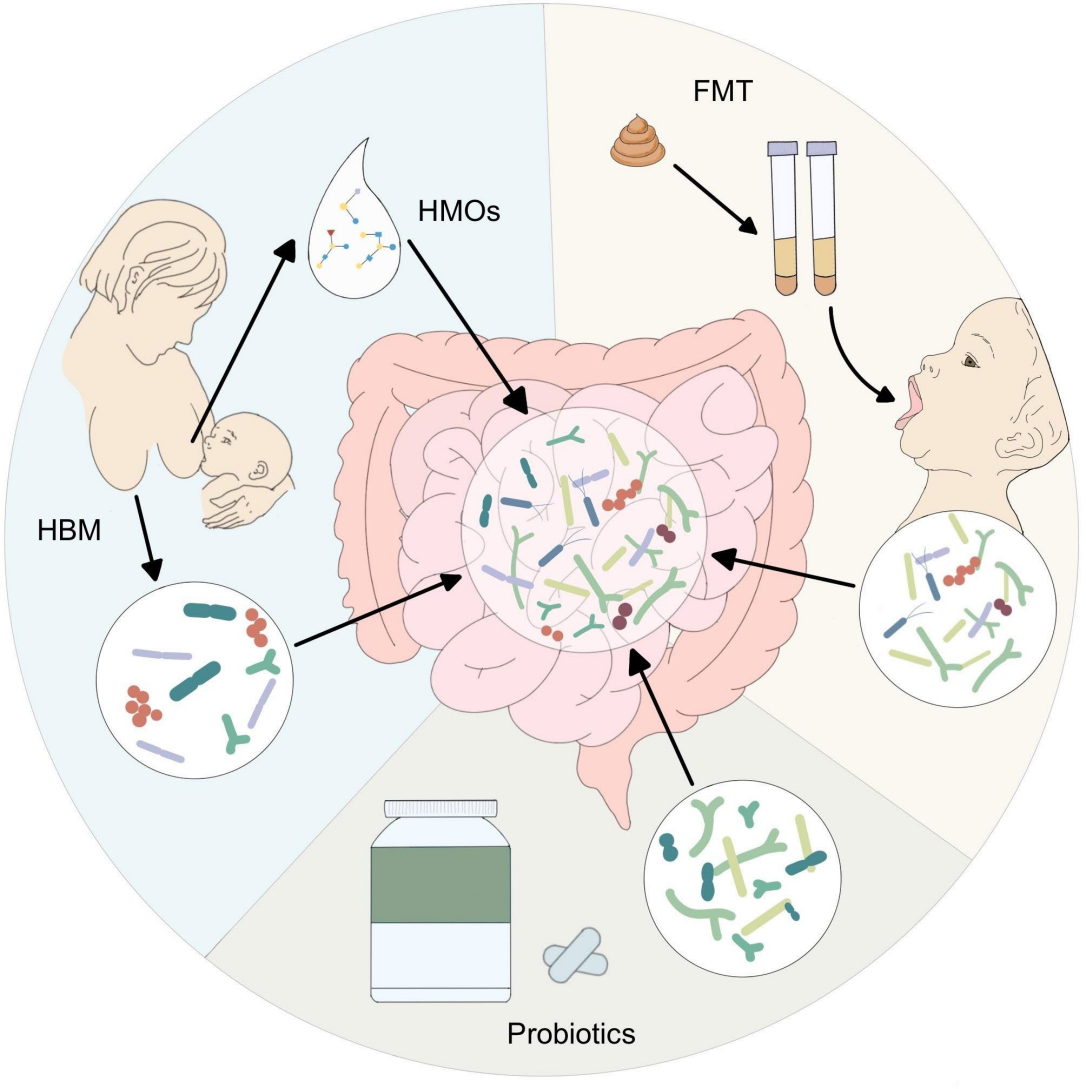
**Potential strategies for improving dysbiosis in preterm infants (PTIs).** The human milk microbiome and human milk oligosaccharides (HMOs) can effectively protect the gut microbiota (GM) in PTIs. Probiotics administration and fecal microbiota transplantation (FMT) can be used to improve preterm intestinal dysbiosis

Intestinal dysbiosis in PTIs affects normal intestinal function and can threaten the life of PTIs (Weiss and Hennet 2017; Graspeuntner et al. 2019; Thänert et al. 2021). However, the exact mechanism underlying dysbiosis in the premature gut is not completely understood. Many factors help shape the preterm GM, such as delivery mode, antibiotic use, and feeding type. In this review, we provide an overview of the development of the preterm GM and summarize the microbial differences associated with contributing factors (Table 1). We also discuss two promising strategies to protect against dysbiosis, human breast milk (HBM) feeding and probiotics administration (Fig. 1).

**Table 1:** Alterations in the gut microbiota of preterm infants related to different factors

Factors	Alterations	Reference
**PROM** ^**a**^ **and chorioamnionitis**	↑ S*taphylococcus* ↑ *Streptococcus* ↑ *Serratia* ↑ *Parabacteroides*	Chernikova, et al. (2016)
**Pre-eclampsia**	↓ *Escherichia/Shigella*	Westaway, et al. (2021)
**GDM**	↑ Firmicutes ↓ Alpha-diversity ↓ Proteobacteria ↓ *Prevotella* ↓ *Lactobacillus*	Chen, et al. (2021), Su, et al. (2018)
**C-section**	↑ Firmicutes ↑ Actinobacteria ↑ *Clostridium sensu stricto* ↓ *Bacteroides*	Pammi, et al. (2017), Rutayisire, et al. (2016), Gregory, et al. (2015), Hill, et al. (2017)
**Prenatal antibiotic**	↓ *Bifidobacterium*	Zou, et al. (2018)
**IAP**	↓ Alpha-diversity ↓ Bacteroidetes ↑ Proteobacteria ↑ *Bifidobacteria* ↑ *Staphylococcaceae* ↑ Unclassified bacilli ↓ *Enterobacteriaceae* ↑ *Comamonadaceae*	Arboleya, et al. (2015), Diamond, et al. (2021), Dierikx, et al. (2020)
**Postnatal antibiotic (PTIs)**	↑ *Enterococcus* ↓ *Bifidobacteria* ↓ *B. fragilis* ↓ Bacteroidetes	Zou, et al. (2018), Penders, et al. (2006), Chang, et al. (2021)
**MOM**	↑ Alpha-diversity ↑ *Bacteroides* ↑ *Bifidobacterium* ↑ *Enterococcus*	Ford, et al. (2019), Gregory, et al. (2016)

^a^PTIs, preterm infants; PROM, premature rupture of the fetal membrane; GDM, gestational diabetes mellitus; C-section, cesarean section; IAP, Intrapartum antibiotic prophylaxis; MOM, mother’s own milk

### Two hypotheses: “sterile womb” and “*in-utero* microbial colonization”

The human womb has always been considered sterile (Sterpu et al. 2021), and multiple studies have indicated that the placenta and amniotic fluid are devoid of microbiota (Leiby et al. 2018; Li et al. 2020). However, in some cases, small amounts of bacterial DNA have been detected in the placenta, fetal tissues, and amniotic fluid using high-throughput molecular technologies. Considering that prenatal factors can influence gut microbial colonization early in life (Chernikova et al. 2016; Chen et al. 2021; Westaway et al. 2021), many scientists have challenged the concept of a “sterile womb” and have suggested that gut microbial colonization in a healthy state may begin *in utero*. Studies addressing these two hypotheses have been conducted recently (Li et al. 2020; Stout et al. 2013; Younge et al. 2019; Goffau et al. 2019; Sharlandjieva et al. 2023), and their findings are summarized in Table 2.

**Table 2:** Research studies related to the two hypotheses: “sterile womb” vs. “*in-utero* microbial colonization”

Reference	Region	Samples	Study subjects (n)	Methods	Major result	Support for hypothesis
Sterpu, et al. (2021)	Sweden	Three layers of placental tissue; amniotic fluid; vernix caseosa; and saliva, vaginal, and rectal samples	76	PCR, DNA sequencing techniques	No evidence to support the existence of a placental microbiome	Sterile womb
Leiby, et al. (2018)	USA	Placental samples	40 (20 term and 20 preterm)	16 S rRNA, shotgun metagenomics	No evidence to support the existence of a placental microbiome	Sterile womb
Li, et al. (2020)	USA	Fetal intestine	-	16 S rRNA	Did not detect any bacterial DNA	Sterile womb
Stout, et al. (2013)	USA	Different regions of the placenta	159 (127 term and 68 preterm)	Histological staining	Evidence of intracellular bacteria in the basal plate of the placenta in 27% of cases	*In-utero* microbial colonization
Younge, et al. (2019)	USA	Human: endometrial surface (uterus), placenta, and amniotic membrane; Mice: fetal intestine	Human: 10 (5 term and 5 preterm)	16 S rRNA gene sequencing, fluorescence in situ hybridization, and bacterial culturing	Bacterial 16 S rDNA signatures were identified in the placentas of women; *Lactobacillus* and other microbes were present in murine fetal tissues	*In-utero* microbial colonization
de Goffau, et al. (2019)	UK	Placental samples	537 (318 cases of adverse pregnancy outcome, 219 controls )	16 S rRNA, shotgun metagenomics	The human placenta does not have a microbiome	Sterile womb
Sharlandjieva, et al. (2023)	Canada	Placental villi, maternal decidua, and fetal embryonic organ tissues	25	16 S rRNA gene sequencing	Failed to identify placental microbiota	Sterile womb
Seferovic, et al. (2019)	USA	Placental tissue	52 (26 term and 26 preterm)	In situ hybridization, traditional histological methods, clinical culture methodologies	Placental microbes were detected by in situ hybridization	*In-utero* microbial colonization
Aagaard, et al. (2014)	USA	Placental specimens	320	16 S rDNA and whole-genome shotgun sequencing and analysis	Placenta harbored a unique low-abundance microbiome	*In-utero* microbial colonization
Theis, et al. (2020a)	USA	Rhesus macaques: fetal and placental samples, uterine wall	Rhesus macaques: 4	Culturing, qPCR, and 16 S rRNA gene sequencing	No existence of a placental microbiota	Sterile womb
Theis, et al. (2020b)	USA	Mice: fetal and placental samples	Mice: 11	Culturing, qPCR, and 16 S rRNA gene sequencing	No consistent evidence for placental and fetal microbiota in mice	Sterile womb

In a cross-sectional study of 195 patients, 27% showed intracellular bacteria in their placental basal plate (Stout et al. 2013). In another study of full-term and unlabored cesarean deliveries, placental microbes were detected by in situ hybridization, but they could not be visualized using traditional histological or clinical culture methodologies (Seferovic et al. 2019). Younge et al. (2019) described the presence of bacterial DNA and viable bacteria in the *in-utero* environment of humans and mice, and suggested that the placenta may be an important source of microbiota in both organisms. Aagaard et al. (2014) collected 320 placental specimens and characterized a unique, but low-abundance, placental microbiome composed of nonpathogenic commensal microbiota similar to the oral microbiota. Amanda et al. (Prince et al. 2016) extracted DNA from placental membranes and found oral and urogenital commensals, such as *Fusobacterium* spp. and *Streptococcus thermophilus.* Therefore, researchers speculated that the placental microbiome may be established by the hematogenous spread of the maternal oral microbiota (Aagaard et al. 2014; Han et al. 2006, 2010; Fardini et al. 2010).

Previous studies, however, could not adequately detect low-biomass microbial populations and lacked appropriate controls against contamination. Sharlandjieva et al. (2023) hypothesized that the abundance of placental microbiota might be related to placental perfusion by analyzing placental villi, maternal decidua, and dental embryonic organ tissues from 5 to 19 weeks of gestation age (GA). However, their observations did not support the existence of an apparent placental microbiome in early pregnancy, let alone support their hypothesis (Sharlandjieva et al. 2023). There was no overlap between the bacterial DNA detected in the different sequencing studies, and the low-abundance and low-biomass microbiota seemed far from being able to initiate “fetus colonization.” A recent study involving 537 women (318 with adverse pregnancy outcomes and 219 controls) found extremely small amounts of bacterial DNA, the majority of which was identified as contamination from laboratory reagents and equipment (Goffau et al. 2019). In another study, fetal intestines were obtained from electively terminated fetuses at 14–23 weeks of gestation, and no bacterial DNA was detected (Li et al. 2020). Furthermore, in other animal experiments, there was no evidence of microbial communities in the fetal and placental tissues of rhesus macaques (Theis et al. 2020a, b) and mice (Theis et al. 2020a, b). Thus, support for the “*in-utero* microbial colonization” hypothesis requires more high-quality evidence. However, investigation of the fetal microbiome remains challenging because of the non-culturable content, risks associated with invasive testing of the fetus, and potential contamination (Perez-Muñoz et al. 2017). As a result, current opinion and support for the “sterile womb” hypothesis remain mainstream (Leiby et al. 2018; Theis et al. 2019).

### Evolution of the gut microbiota in PTIs

High-throughput molecular methods help us further understand the details of the GM (Liu et al. 2021). Firmicutes, Bacteroidetes, Actinobacteria, and Proteobacteria are the major phyla found in the gut of healthy adults. It is generally accepted that neonates experience normal initial colonization of microbiota from the maternal vagina and rectum during vaginal birth. Neonates delivered via cesarean section (C-section) carry bacteria from the skin of healthcare professionals and/or the environment. After interaction with the maternal microbiota, multiple factors contribute to initial colonization and GM development, including preterm birth, feeding type, antibiotic therapy, and probiotics (Collado et al. 2012). During the first few days of life, *Bifidobacterium* and *Enterobacteriaceae* dominate in the gut of FTIs (Eggesbø et al. 2011; Bokulich et al. 2016), but from day 10 to 3 months of age, *Bifidobacterium* and *Bacteroides* dominate (Arboleya et al. 2012).

Gut microbial colonization of PTIs differs significantly from that of FTIs in displaying less diversity, delayed colonization by *Bifidobacteria*, and more opportunistic and potential pathogen growth, including that of *Enterococcus*, *Staphylococcus*, and *Enterobacter*, during early life (Itani et al. 2017). In the first week of life, GM diversity in PTIs is low (Drell et al. 2014), with colonization by facultative bacteria, such as *Enterobacteriaceae* (Younge et al. 2019), *Streptococcus*, *Enterococcus*, and *Staphylococcus* (Bokulich et al. 2016; Itani et al. 2017; Drell et al. 2014). With increasing postmenstrual age (PMA) among PTIs fed human breast milk (HBM), the GM switches from one dominated by *Staphylococcus* and *Enterococcus*, to one dominated by *Enterobacter*, and finally towards *Bifidobacterium*-dominated anaerobic genera, such as *Bacteroides* and *Clostridium* (Drell et al. 2014; Korpela et al. 2018). Diversity increases over 2 months (Drell et al. 2014). Regardless of the gestational age at birth, infants begin to proceed towards a *Bifidobacterium*-dominated GM composition, an indicator of a healthy microbiota, after 30 weeks of PMA (Korpela et al. 2018).

**Fig. 2:**
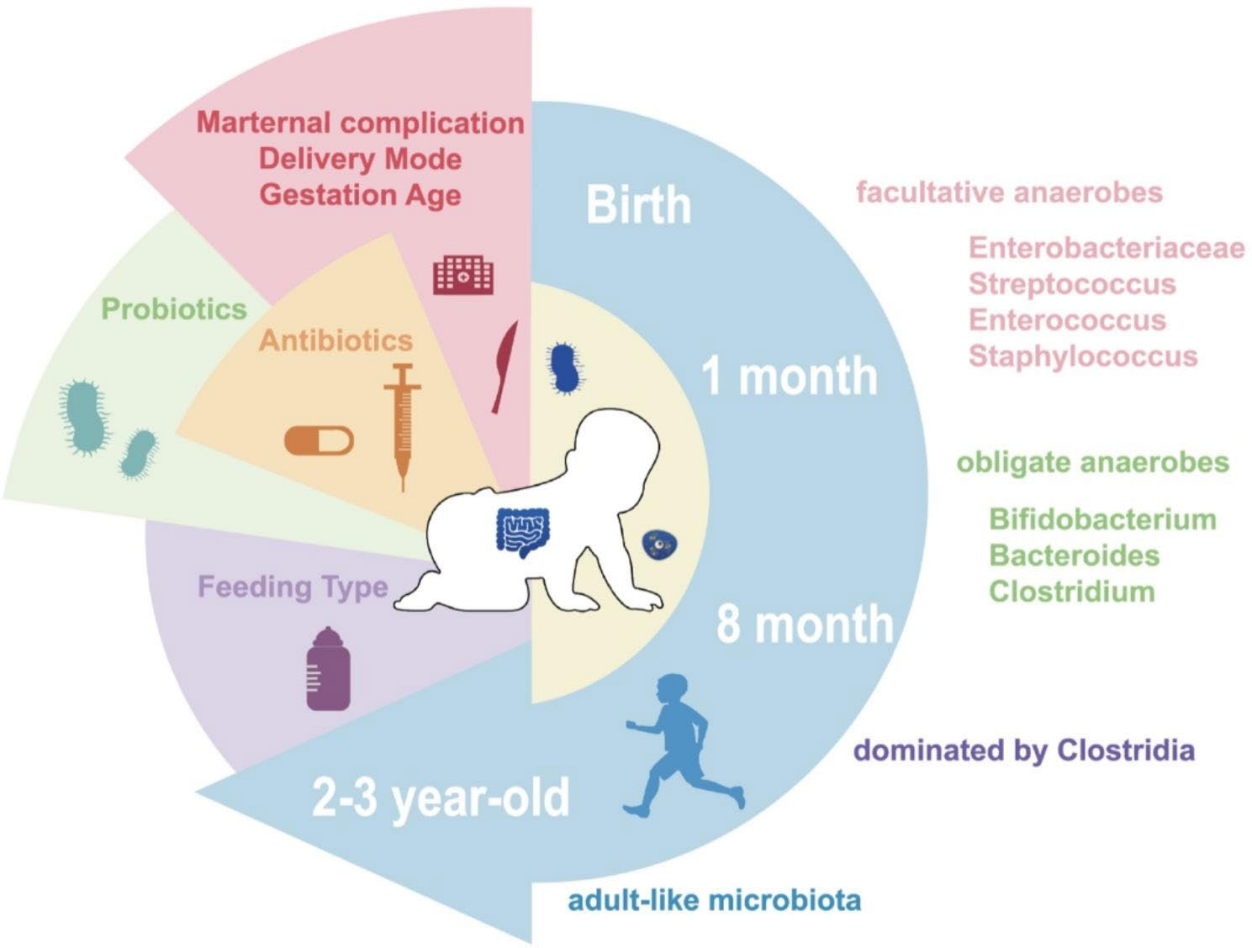
**Factors shaping the preterm infant (PTI) gut microbiota during early life and evolution** During the first weeks after birth, the human infant gut is colonized by facultative anaerobes, such as *Enterobacteriaceae*, *Streptococcus*, *Enterococcus*, and *Staphylococcus*. PTIs are more highly colonized by *Staphylococcus* than are full-term infants (FTIs) during this period, and PTIs gradually become dominated by anaerobic genera, including *Bifidobacterium*, *Bacteroides*, and *Clostridium*. Between 10 days and 3 months of age, *Enterococcaceae* and *Lactobacilli* dominance is observed in premature babies. After cessation of breastfeeding and the addition of solid foods, the gut microbiota gradually becomes dominated mainly by members of the anaerobic class Clostridia, a process required for maturation into an adult-like microbiota

When infants (both PTIs and FTIs) are weaned (Oyedemi et al. 2022), the GM gradually becomes dominated by anaerobic *Clostridia* (Bäckhed et al. 2015). The cessation of breastfeeding affects microbial composition and function more significantly than does the addition of solid food (Oyedemi et al. 2022). This process is crucial for transformation into an adult-type microbiota (Bäckhed et al. 2015; Palmer et al. 2007). By approximately 2 years of age, the GM of children resembles that of adults (Bokulich et al. 2016). Serious diseases (e.g., NEC or LOS), exposure to antibiotics, and C-section may have no significant long-term effects on the GM of PTIs (Stewart et al. 2015) (Fig. 2).

### Maternal conditions

The prevailing view is that before birth, the fetus develops in a sterile environment. The presence of bacteria in the amniotic cavity and fetal membranes is often associated with preterm delivery. Exposure to a nonsterile intrauterine environment aggravates the aberrant initial colonization of the GM induced by preterm birth (Chernikova et al. 2016; Westaway et al. 2021; Roswall et al. 2021). A prospective longitudinal study found that during hospitalization, PTIs exposed to premature rupture of fetal membranes and chorioamnionitis had a higher abundance of intestinal pathogenic bacteria (including *Staphylococcus*, *Streptococcus, Serratia*, and *Parabacteroides*) than that of non-exposed PTIs, irrespective of postnatal antibiotics (Chernikova et al. 2016). Westaway et al. (2021) also reported significantly higher *Staphylococcus* gut colonization in PTIs with maternal chorioamnionitis at admission; however, these changes were not permanent (Chernikova et al. 2016; Westaway et al. 2021).

In contrast, infants whose mothers experienced non-infectious pregnancy complications, such as gestational diabetes mellitus (GDM) or preeclampsia, exhibited a different GM from that of healthy controls (Chen et al. 2021; Westaway et al. 2021; Roswall et al. 2021). Preeclampsia and GDM influence the maternal GM (Chen et al. 2020; Li et al. 2022). Although the exact mechanism of gut microbial transfer from mother to fetus is unknown, it is possible that the GM of neonates follows maternal alterations (Collado et al. 2012; Jost et al. 2014; Hiltunen et al. 2021; Valles-Colomer et al. 2023). At discharge, *Escherichia/Shigella* was significantly lower in the gut of PTIs whose mothers were diagnosed with preeclampsia (Westaway et al. 2021). Chen et al. (2021) observed that the abundances of *Firmicutes* and *Proteobacteria* changed significantly and alpha diversity decreased in neonates of mothers with GDM. Moreover, the relative abundances of *Prevotella* and *Lactobacillus* were also significantly lower (Su et al. 2018). However, 5 years after delivery, the abundance of the top 15 genera and alpha diversity were similar between the GDM and non-GDM groups, in both women and neonates, in a cross-sectional study of 237 subjects (Hasan et al. 2018). Notably, there remained a similar microbiome composition between a mother and her own child when compared with that of others.

The maternal GM is relevant to immune development in infants, neurodevelopment in children, and the development of asthma (Gomez et al. 2016; Macpherson et al. 2017; Sun et al. 2023). After birth, mother-to-infant gut microbial transmission is indispensable for establishing the infant GM (Ferretti et al. 2018). During infancy, this transmission is considerable and stable, and shared species comprise approximately half of the same strains (Valles-Colomer et al. 2023). In one case report by Wei et al. (2022), a pregnant patient infected with *Clostridioides difficile* received a fecal microbiota transplantation (FMT), and demonstrated the cross-generational transfer of donor fecal bacteria to her late-born infant. Intervention with the maternal GM before labor may be a novel strategy for modulating the infant GM, especially when the mother is experiencing gut dysbiosis.

### Delivery mode

Data from 154 countries covering 94.5% of live births showed that 21.1% of women gave birth via C-section in the past decade (Betran et al. 2021). Premature births accounted for 50% of the C-Sect. (Bannister-Tyrrell et al. 2015). During a C-section birth, the mother-to-neonate microbial colonization is disturbed owing to limited vertical transmission (Liu et al. 2015). Most studies on the impact of delivery mode on the GM have focused on FTIs, and indicated that infants delivered by C-section bypass the vaginal seeding process and thus develop an abnormal GM (Korpela et al. 2018; Madan et al. 2016; Rutayisire et al. 2016; Shao et al. 2019; Selma-Royo et al. 2020). In these cases, the GM of neonates is dominated by skin bacteria (e.g., *Staphylococcus* and *Streptococcus*) from the environment (Korpela et al. 2018). Opportunistic pathogens from hospital environments, including *Enterococcus*, *Enterobacter*, and *Klebsiella* spp., pose a significant risk of future infection (Shao et al. 2019). *Lactobacillus* spp., which mainly come from the maternal vagina, colonize the gut later and weaker in infants delivered by C-Sects. (Nagpal et al. 2016; Kervinen et al. 2019). Disrupted transmission of maternal *Bacteroides* strains has also been reported (Shao et al. 2019; Nagpal et al. 2016; Kervinen et al. 2019). Rutayisire et al. (2016). indicated that the influence of delivery mode on the GM of FTIs disappears at approximately 6 months of age.

Compared to FTIs, PTIs are more likely to receive antibiotic treatment and hospital care, which may shape the development of their GM. The impact of delivery mode is also confounded by prematurity. After adjusting for these factors, the delivery mode was still shown to affect the GM in some studies. For example, compared with the other delivery mode at the phylum level, the relative abundance of *Firmicutes* was higher in PTIs born via C-section, whereas the abundance of *Bacteroidetes* was higher in PTIs born via vaginal delivery (Pammi et al. 2017). Additionally, a lower abundance and diversity of *Actinobacteria* were associated with C-section delivery in infants from birth to 3 months of age (Rutayisire et al. 2016). At the family/genus level, the abundance of *Bifidobacterium* and *Bacteroides* increased significantly over time among vaginally delivered infants and they were not influenced by antibiotic administration or nutritional factors (Gregory et al. 2015). Moreover, these genera in vaginally delivered infants were significantly more constant than in those born via C-Sect. (Rutayisire et al. 2016). The prevalence and abundance of *Lactobacillus* were similar between infants delivered vaginally or by C-Sect. (Shao et al. 2019), whereas *Bacteroides* colonization was significantly delayed in infants delivered via C-Sect. (Gregory et al. 2015). *Clostridium sensu stricto* was more abundant in PTIs born via C-section than in PTIs delivered vaginally during the first week of life (Hill et al. 2017). The delivery mode had a minimal effect on *Bacteroides* colonization by the age of 6–12 months (Rutayisire et al. 2016).

However, current research is not univocal regarding the influence of the delivery mode on the GM. In previous studies, the delivery mode did not correlate with detectable differences in the composition of the GM between preterm groups on day 7 (Patole et al. 2016; Esaiassen et al. 2018). Hill et al. (2017) compared the GM of PTIs (C-section, n = 35; vaginal birth, n = 4) at the same age from 1 to 24 weeks after birth and found no difference in the relative proportion of *Bifidobacterium* at any time point. This finding is consistent with the results of another study (Imoto et al. 2021). During the first 3–4 days postpartum, no differences in GM composition were observed using 16 S rRNA gene profiling in infants delivered by the two different modes (Hiltunen et al. 2021). Nonetheless, fecal samples from vaginally delivered infants showed high levels of *Bacteroides* using qPCR analysis on day 10 (Arboleya et al. 2015). Interpretation of the results of the above-mentioned studies may suffer from experimental limitations, such as small sample size, long sampling interval, low detection sensitivity, and lack of association analysis between the maternal microbiota and the preterm GM. Factors, including prenatal conditions, GA, hospital stay, and antibiotic use, can inevitably lead to bias in the analysis and comparison of results.

Normally, the vaginal seeding process plays a crucial role in determining the difference between the two delivery modes. However, in randomized controlled trials, orally administered vaginal bacteria, as a simulated form of vaginal seeding, did not alter the GM of infants born by cesarean Sects. (Butler et al. 2020; Wilson et al. 2021). During the first year of life, there was a significantly lower similarity between the GM of infants born via C-section vs. vaginally, as compared to their respective mothers (Bäckhed et al. 2015). Korpela et al. (2020) found that after oral FMT from mothers to their FTIs, the GM was similar between C-section and vaginally delivered infants. This suggests that the maternal GM, rather than the vaginal microbiota, plays an important role in maternal–neonatal microbial transmission. In the future, novel interventions and therapies to improve the health of PTIs may take advantage of the known transmission from the maternal GM to PTIs.

### Antibiotics

Antibiotic exposure significantly alters the abundance of bacteria and delays microbial maturation and colonization by certain bacterial taxa during the first 2 years of life (Bokulich et al. 2016). Moreover, dysbiosis mediated by antibiotics is associated with NEC, LOS, and other adverse health outcomes (Deshmukh et al. 2014; Zhou et al. 2020). The effects of maternal and PTI exposure to antibiotics are discussed below.

### Maternal exposure to antibiotics

In the full cohort of 1,347,018 infants (live singletons born between 2006 and 2018), 294,657 (21.9%) were exposed to prenatal antibiotics (Nakitanda et al. 2023). Indications for obstetric antibiotics include clinical chorioamnionitis, group B *Streptococcus* infection, premature rupture of fetal membranes, and prophylactic administration for premature birth (Martinez de Tejada 2014; (2018) 2018; (2020) 2020; Ronzoni et al. 2022). Antibiotic exposure (prenatal and postnatal) influences the early establishment of the GM in patients with PTIs (Zou et al. 2018). A higher load of *Lactobacillus* was observed in the meconium of PTIs without antibiotic exposure than in those with perinatal antibiotic exposure (Zhou et al. 2020). The abundance of *Bacteroidetes* and *Bifidobacterium* was significantly decreased 7 and 14 days after birth. Colonization by *Bifidobacterium* was delayed in the prenatal antibiotic-exposure group (Zou et al. 2018). Maternal exposure to antibiotics can disturb the maternal GM, and maternal intestinal dysbiosis may be transmitted to neonates (Nyangahu et al. 2018).

Intrapartum antibiotic prophylaxis (IAP) are frequently administered during emergency C-section. This may result in a decrease in the alpha diversity and abundance of *Bifidobacteria* (Diamond et al. 2021). Dierikx et al. (2020) found a decreased abundance of Bacteroidetes and a concurrent increase in Proteobacteria in the fecal samples of neonates whose mothers had received IAP.

The effects of antibiotics on the establishment of the GM are minimal within the first few days after delivery, becoming more apparent later (Arboleya et al. 2015). At 1 month of age, a higher relative abundance of *Comamonadaceae, Staphylococcaceae*, and unclassified bacilli, as well as a lower relative abundance (P < 0.05) of *Enterobacteriaceae* were observed in PTIs from IAP-exposed mothers than in those from non-IAP-exposed mothers. Most of these differences, however, disappeared at 90 days of age (Arboleya et al. 2015).

### PTI exposure to antibiotics

PTIs are susceptible to bacterial translocation from the gut and other epithelial surfaces into the bloodstream; therefore, prophylactic antibiotic therapy is common for PTIs (Nguyen et al. 2016). The oral administration of antibiotics (mainly amoxicillin) to infants decreases the abundance of *Bifidobacteria* and *B. fragilis* during the first month of life (Penders et al. 2006). The abundance of Bacteroidetes decreases with increasing antibiotic exposure time (Zou et al. 2018). Different drugs exhibit varying effects, e.g., cephalosporins are associated with a slow increase in *Bifidobacterium* over time (Coker et al. 2020). b-lactam antibiotics are associated with a slower increase in several taxa, including *Bacteroides* (Coker et al. 2020) within the first year of life and have a major influence on the *Bifidobacterium* population in newborns. This influence is most significant in 1-month-old infants, persists for 3 months, gradually weakens, and then disappears by approximately 6 months of age (Shao et al. 2019).

Chang et al. (2021) conducted an observational study of 24 breastfed very low birth weight (VLBW) PTIs administered ampicillin-gentamicin (n = 10) or ampicillin-cefotaxime (n = 14). No statistically significant differences were detected in the observed bacterial phyla between the two groups at 7, 14, and 30 days after birth. *Enterococcus* was significantly more abundant in newborns treated with ampicillin-cefotaxime than in those treated with ampicillin-gentamicin, especially on day 7. Excessive growth of *Enterococcus* disappeared in newborns treated with cefotaxime at 1 month of age.

Although antibiotics disrupt the richness and composition of the GM, recent studies have indicated that short-term enteral antibiotics confer benefits to PTIs shortly after birth (Nguyen et al. 2016; Birck et al. 2016). Enteral antibiotics, rather than systemic antibiotics (Nguyen et al. 2016) may help the intestine mature structurally, functionally, and immunologically by delaying microbial colonization and reducing interference from colonized bacteria (Birck et al. 2016; Jensen et al. 2014). Moreover, systemic immunity and resistance to LOS are improved by delayed colonization of the premature gut (Nguyen et al. 2016).

Bokulich et al. (2016). demonstrated that the influence of antibiotics was weaker than that of the delivery mode and age. The duration of antibiotic administration influences the GM for no longer than the first 2 weeks of life (Stewart et al. 2015; Costeloe et al. 2016). Further research is needed to optimize antibiotic exposure and explore whether breastfeeding can minimize the adverse effects of antibiotic exposure (Azad et al. 2016). Timing, mode, duration, drug type, and underlying conditions should be considered for prophylactic antibiotic treatment of PTIs.

### Feeding type

HBM is the primary nutrition choice for all healthy and ill neonates, including PTIs. HBM contains nutritional components, distinct bioactive molecules, and immunological factors (Ballard and Morrow 2013), which provide short- and long-term benefits, including nutritional, immunological, developmental, etc., and may be associated with a decreased risk of NEC when compared with formula-feeding (Leoz et al. 2015; Ford et al. 2019). With the growing knowledge of HBM composition, insight has been gained into the mechanism of protective effects of HBM on PTIs. Human milk oligosaccharides (HMOs) and HBM microbiota play roles in the establishment of the preterm GM (Leoz et al. 2015; Jost et al. 2013; Zehra et al. 2018; Bhowmik et al. 2022). In the absence of the mother’s own milk (MOM), donor human milk (DHM) can also meet nutritional requirements, promote intestinal health, and support resistance against pathogens (Li et al. 2017). DHM must be pasteurized to inactivate potentially harmful viral and bacterial agents. After pasteurization, the relative abundance of *Staphylococcus* decreased, whereas that of *Streptococcus* and *Pseudomonas* increased (Beghetti et al. 2022). Previous data indicated that maternal gut bacteria may influence neonatal gut colonization via the entero-mammary pathway (Jost et al. 2014). However, little is known about whether pasteurization affects the process of passing maternal milk microbiota to infants. Formula milk, which has a high caloric density and protein content, is a good nutritional source when HBM is unavailable (Chinnappan et al. 2021; Moreira-Monteagudo et al. 2022). Currently, research is focused on how feeding patterns influence the outcome and development of the GM in PTIs (Table 3).

**Table 3:** Alterations in the preterm infant gut microbiota related to feeding type

Reference	Region	Sample size (n)			Sample time	Alterations in the GM^a^ of PTIs		
		MOM	DHM	Formula		MOM	DHM	Formula
Ford, et al. (2019)	USA	74	43	-	Within 6 weeks after birth	↑Alpha-diversity		-
					Week 4	↑*Bacteroides*, *Bifidobacterium*, and *Enterococcus*	↑*Staphylococcus*	-
Parra-Llorca, et al. (2018)	Spain	34	28	7	By the time of full enteral feeding	↓*Clostridiaceae*, ↑*Bifidobacterium, Staphylococcus, Clostridium, Serratia, Coprococcus, Aggregatibacter, and Lactobacillus*	↓Actinobacteria, ↑Bacteroidetes	-
							*Acinetobacter* genus was found	Bacteroidetes was highest; *Staphylococcus* and *Klebsiella* were dominant
Gregory, et al. (2016)	USA	10	10	10	First 60 days	Initial increase in diversity		*Lactobacillales* was highest
Wang, et al. (2020)	USA	10	-	10	At an average of 15 and 17 days after birth	*Veillonella*, *Escherichia/Shigella*, *Staphylococcus*, *Clostridium*, *Enterococcus*, and *Streptococcus were dominant*	-	↓*Proteobacteria*

^a^GM, gut microbiota; PTIs, preterm infants; MOM, mother’s own milk; DHM, donor human milk

### HBM microbiome may bridge the maternal GM and the GM of offspring

**Fig. 3:**
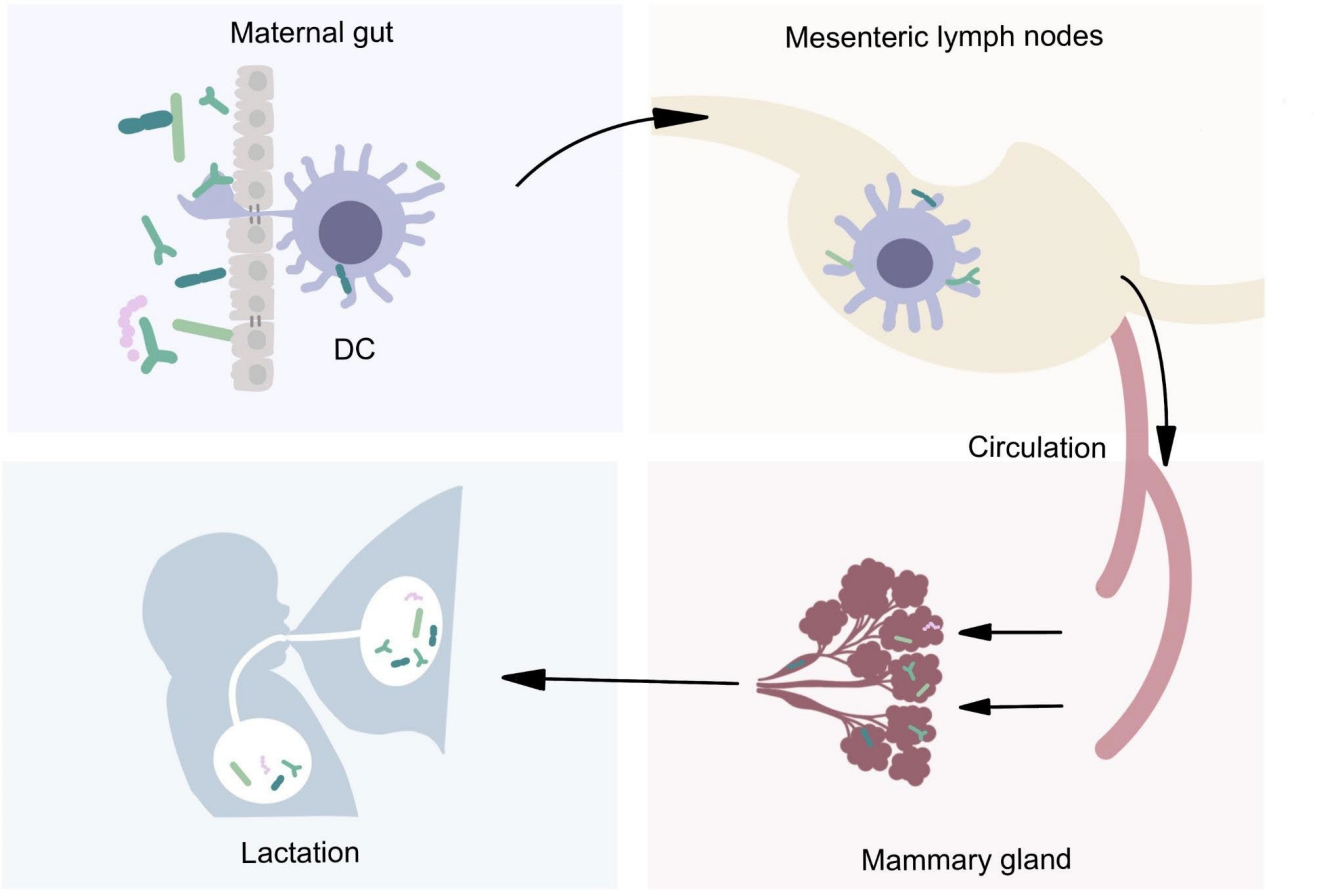
**Entero-mammary pathway** Dendritic cells send dendrites out of the epithelium through tight junctions. Dendritic cells carrying bacteria migrate to the mesenteric lymph nodes, lactate mammary glands, and ultimately into milk. Through this entero-mammary pathway, the maternal gut microbiota (GM) finally reaches the gut of preterm infants (PTIs)

HBM contains a highly diverse and complex microbiome (Jost et al. 2013) that may help establish the infant GM. The HBM microbiome affects the colonization of the GM of PTIs, including with beneficial, commensal, and potentially probiotic bacteria (Yi and Kim 2021), and it can be influenced by antibiotics (Fernández et al. 2020). An analysis of 16 subjects demonstrated that there is a “core” microbiome in HBM composed of nine operational taxonomic units, including *Staphylococcus*, *Streptococcus*, *Serratia*, *Pseudomonas*, *Corynebacterium*, *Ralstonia*, *Propionibacterium*, *Propionibacterium*, *Sphingomonas*, and *Bradyrhizobiaceae* (Hunt et al. 2011). The GM can be transmitted vertically from mother to infant via lactation (Jost et al. 2014; Valles-Colomer et al. 2023; Zhong et al. 2022), and the HBM microbiota may originate from the maternal gastrointestinal tract (Greiner et al. 2022). Dendritic cells send dendrites out of the epithelium via tight junctions (Rescigno et al. 2001), and dendritic cells carrying bacteria migrate to the mesenteric lymph nodes (Macpherson and Uhr 2004), lactate mammary glands, and ultimately into milk (Greiner et al. 2022; Perez et al. 2007). This process is known as the entero-mammary pathway (Fig. 3).

### HMOs promote the growth of “good bacteria” and inhibit pathogenic colonization

**Fig. 4:**
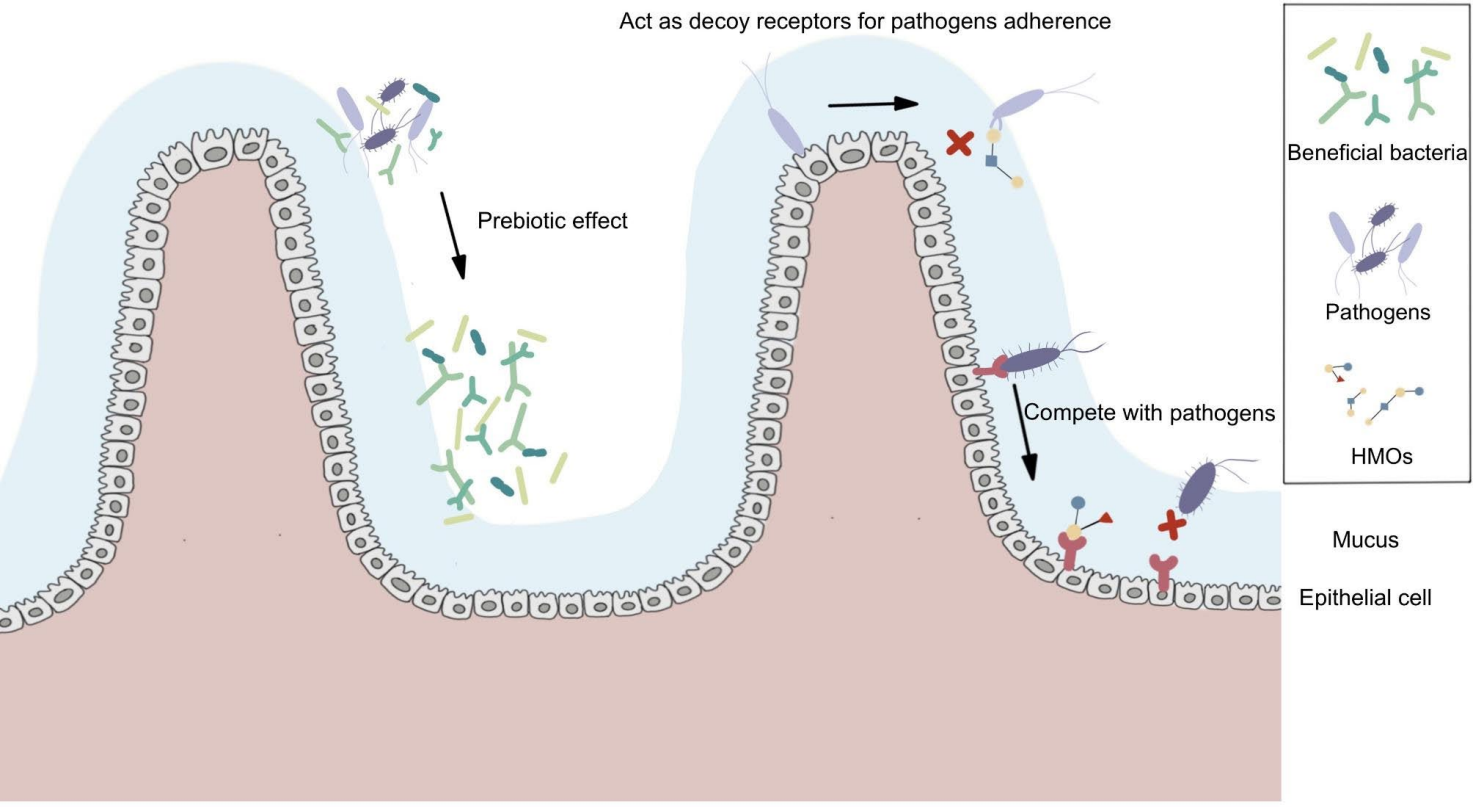
**Human milk oligosaccharides (HMOs) promote the growth of “good bacteria” and inhibit pathogenic colonization** HMOs help establish a healthy gut microbiota (GM) in at least two ways. First, HMOs exhibit a probiotic effect, promoting the growth of beneficial bacteria and inhibiting the growth of pathogens. Second, HMOs act as decoy receptors and bind pathogens, competing with them through adhesion to their receptors on epithelial cells, suppressing the colonization of pathogenic bacteria

HMOs, which are nondigestible carbohydrates, are the third largest solid component in human milk and are highly variable and unique (Aakko et al. 2017). It is well-established that the probiotic and immunomodulatory function of HMOs can help promote intestinal maturation and barrier function (Zehra et al. 2018; Bhowmik et al. 2022; Goehring et al. 2016). HMOs help establish a healthy GM in at least two ways. First, they exhibit probiotic effects and selectively promote the growth and colonization of beneficial bacteria, including *Bifidobacterium* and *Bacteroides* (Marcobal et al. 2011). Second, HMOs suppress the growth and colonization of pathogenic bacteria. HMOs function as anti-adhesive molecules by acting as decoy receptors to bind pathogens and inhibit their colonization (Newburg et al. 2005; Shoaf-Sweeney and Hutkins 2009). HMOs compete with pathogens for adhesion to carbohydrate receptors on epithelial cells, further preventing the adhesion of pathogens to their receptors (Angeloni et al. 2005; Coppa et al. 2006; Weichert et al. 2013) (Fig. 4).

A proof-of-concept study proved that HMOs selectively enrich the growth of beneficial bacteria, including *Bifidobacterium* and *Bacteroides* (Marcobal et al. 2011). In a large-scale study of 1023 infants, HMOs showed natural variations and influenced the GM of infants (Barnett et al. 2023). Lacto-N-hexaose and 6′-sialyllactose were positively and negatively associated with the abundance of *Bifidobacterium*, respectively (Barnett et al. 2023). Additionally, the variable composition of HMOs can be explained by maternal genotype, including the secretor (FUT2) and Lewis (FUT3) genes, which, notably, do not drive major differences in the GM between infants (Barnett et al. 2023). These results suggest various mechanisms related to HMOs are involved and that further research is needed.

Clinical trials have investigated the effects of HMO interventions on the GM in PTIs and other animals (Torres Roldan et al. 2020; Wang et al. 2020a, b). Shortly after birth, the primary microbial consumers of HMOs (*Bifidobacteria* and *Bacteroidetes*) are not dominant in the preterm gut (Barnett et al. 2023). HMO supplementation of formula did not aid in the maturity of the immature intestine or prevent NEC in preterm pigs during the first weeks of life, and the effects depended on the different stage of intestinal maturity (Cilieborg et al. 2017). Therefore, when the gut reaches a more mature phase and the abundance of HMO-consuming *Bacteroidaceae* and *Bifidobacteriaceae* increases (Barnett et al. 2023), HMOs may exert more protective effects on the gut. Thus, the timing of HMO supplementation in DHM- or formula-fed PTIs should be carefully evaluated (Bering 2018).

### DHM can promote a somewhat similar GM in PTIs as that observed in MOM-fed PTIs

MOM and DHM can provide PTIs with more benefits for microbial colonization than that provided by formula-feeding. However, the GM composition differs significantly between PTIs fed MOM and DHM (Parra-Llorca et al. 2018). Among PTIs fed MOM, a significantly higher alpha diversity and relative abundance of *Bacteroides* were observed within 6 weeks after birth, and a significant increase in the abundance of *Bacteroides*, *Bifidobacterium*, and *Enterococcus* was observed by week 4 of life (Ford et al. 2019). When full enteral feeding (defined as ≥ 150 cc/kg/day of MOM, DHM, or formula) was administered, there was a lower abundance of Actinobacteria (Parra-Llorca et al. 2018) and a higher abundance of Bacteroidetes in the GM of PTIs fed DHM than in those fed MOM alone. The relative abundance of *Bifidobacterium* was higher and that of *Clostridiaceae* was lower after MOM feeding than after DHM feeding (Parra-Llorca et al. 2018). High *Enterobacter* abundance was observed in the GM of the MOM-fed group when full enteral feeding was achieved (Cong et al. 2017), as well as that of *Staphylococcus*, *Clostridium*, *Serratia*, *Coprococcus*, *Aggregatibacter*, and *Lactobacillus*, when compared to the DHM-fed PTIs (Parra-Llorca et al. 2018). Supplementation with pasteurized DHM partially promoted a microbiota similar to that of MOM-fed PTIs, and a moderately rapid increase in bacterial diversity was observed (Gregory et al. 2016).

### Formula feeding induces different microbial patterns in PTIs than that observed in MOM-fed PTIs

MOM-fed PTIs exhibit a higher initial microbial diversity with a more gradual acquisition than observed in formula-fed PTIs (Gregory et al. 2016). In a cohort of 20 PTIs fed MOM or formula, the alpha diversity was similar at 15 and 17 days after birth, but the beta diversity showed a significant difference in composition between groups (Wang et al. 2020a, b). Firmicutes were dominant in both groups, whereas *Veillonella*, *Escherichia/Shigella*, *Staphylococcus*, *Clostridium*, *Enterococcus*, and *Streptococcus* were the dominant members of the GM in MOM-fed PTIs (Wang et al. 2020a, b). *Staphylococcus* and *Klebsiella* were dominant in the gut of formula-fed PTIs, followed by *Enterococcus*, *Clostridium*, and *Veillonella. Peptostreptococcaceae*, a family of gram-positive bacteria in the class Clostridia, was observed only in the formula-fed PTIs, whereas *Acinetobacter* was found only in the DHM-fed PTIs (Parra-Llorca et al. 2018). Formula-fed infants had the highest abundance of *Lactobacillales* (Gregory et al. 2016) and Bacteroidetes (Parra-Llorca et al. 2018) among all feeding types, and a significantly lower abundance of *Proteobacteria* than that of the MOM-fed group (Wang et al. 2020a, b). Cai et al. (2019) indicated that feeding type significantly affects the GM structure at the late feeding stage (2–4 weeks after birth), but not in the early feeding stage (within 2 weeks of birth).

### Probiotics

Probiotics are defined by the WHO as live microorganisms that when administered in adequate amounts, confer health benefits to the host (Morelli and Capurso 2012). The lactic acid-producing genera *Lactobacillus* (including *L. acidophilus* and *L. rhamnosus*) and *Bifidobacterium* (including *B. bifidum*, *B. animals* subsp. *lactis*, and *B. longum* subsp. *infantis*) are the most frequently used probiotic bacteria in humans to manage dysbiosis, followed by *Streptococcus*, *Enterococcus*, *Lactococcus*, *Pediococcus*, *Bacillus*, *Escherichia*, and certain *Saccharomyces* yeast strains (Tanaka et al. 2019; Koutsoumanis et al. 2020, 2022). Probiotics have been shown to comprehensively influence host health in both human and animal studies. Among PTIs, there is increasing evidence that probiotics are effective in promoting health and improving adverse outcomes (Tanaka et al. 2019; Oncel et al. 2014). The benefits include normalizing aberrant GM, reducing microbiota-associated diseases, and improving outcomes in fragile neonates. Alterations in the GM using probiotic therapies are often transient, but in early life stages, especially in the neonatal stage of PTIs, rectifying the aberrant GM in the short term can bring non-negligible benefits.

Many large multicenter studies and placebo-controlled randomized trials have provided evidence that the use of probiotic prophylaxis can prevent NEC and sepsis (Oncel et al. 2014), shorten hospital stays, and reduce overall mortality (Lau and Chamberlain 2015; Dermyshi et al. 2017; Sun et al. 2017). However, the efficacy of probiotics appears to depend on the bacterial strain used in the trials (Costeloe et al. 2016). In the section that follows, we review the impacts of the most promising and common probiotic strains on the premature gut and briefly summarize the potential mechanisms of various probiotics, especially those used in the prevention or treatment of NEC and LOS (Table 4).

**Table 4:** Studies on the use of probiotics and microbiota outcomes

Reference	Type of probiotic	Sample size (n)		Sample time	Outcomes in microbiota samples of PTIs^a^ exposed to probiotics		
		Probiotics	Non-probiotics		Higher abundance	Lower abundance	Other findings
Esaiassen, et al. (2018)	*Lactobacillus acidophilus*, *Bifidobacterium longum* subsp. *infantis*	31	45	Day 7	*Bifidobacterium* and *Lactobacillus*	-	-
Horigome, et al. (2021)	*Bifidobacterium breve* M-16 V	12	10	2–9 weeks after hospital discharge	Actinobacteria, *Bifidobacterium breve* M-16 V, *Bifidobacterium*	Proteobacteria	-
Millar, et al. (2017)	*Bifidobacterium breve* strain BBG-001	40	48	36 weeks post-menstrual age	-	-	No difference in the microbial richness and diversity
Nguyen, et al. (2021)	*Bifidobacterium longum* subsp. *infantis* EVC001	31	46	Throughout hospital stay	*Enterobacteriaceae* and/or *Staphylococcaceae*	-	Total *Bifidobacteriaceae* developed rapidly
Plummer, et al. (2018)	*Bifidobacterium longum* subsp. *infantis* BB-02, *Streptococcus thermophilus* TH-4, *Bifidobacterium animalis* subsp. *lactis* BB-12	38	28	During probiotic administration	*Bifidobacterium*	*Enterococcus*	-
Martí, et al. (2021)	*Lactobacillus reuteri* DSM 17,938	54	54	During first week	*-*	*Staphylococcacea; Enterobacteriaceae*	-
				1–36 weeks PMA	*L. reuteri* DSM 17,938	*-*	-
				During first month	*-*	*-*	Significantly higher bacterial richness, diversity, and evenness
				2 years	*-*	*-*	No significant differences in the gut microbiota
Abdulkadir, et al. (2016)	*Lactobacillus acidophilus-*NCIMB701748, *Bifidobacterium bifidum-*ATCC15696	7	3	During probiotic administration	*Lactobacillus* spp. (highest abundance); *Bifidobacterium*	*-*	Significantly lower Shannon diversity
				After probiotic administration	*Lactobacillus* spp. (highest abundance)	*-*	-

^a^PTIs, preterm infants; PMA, postmenstrual age

### Commonly used probiotic strains

#### Bifidobacterium breve strain BBG-001

A multicenter randomized controlled phase 3 trial (PiPS trial) (Costeloe et al. 2016) showed that formula supplemented with *B. breve* strain BBG-001 did not affect the incidence of LOS, NEC, or death in PTIs. To further explore how probiotics influence the GM of PTIs, another research (Millar et al. 2017) examined 88 fecal samples (48 placebo and 40 probiotics-treated) at 36 weeks PMA and found no statistically significant difference in microbial richness or diversity between groups. Additionally, no probiotic-associated adverse events were recorded (Costeloe et al. 2016).

### Bifidobacterium breve M-16 V

*B. breve* M-16 V presents in the healthy gut. When added to infant formula, it can promote early gut microbial colonization and help regulate the immune balance and inflammatory responses. This strain can protect high-risk infants from allergies and prevent NEC (Wong et al. 2019)development (Wong et al. 2019) by normalizing toll-like receptor (TLR) 4 expression and enhancing TLR2 expression to suppress inflammatory responses, as evidenced in rat models (Satoh et al. 2016).

In one study, LBW infants (n = 22) were either administered *B. breve* M-16 V from birth until hospital discharge (n = 12) or left untreated as controls (n = 10). No significant difference was observed in alpha diversity between gorups (Horigome et al. 2021). The relative abundances of *Bifidobacterium* and *Enterococcus* were significantly higher, whereas those of *Rothia*, *Lactococcus*, and *Klebsiella* were significantly lower in the M-16 V-treated group than in the controls. The abundances of *Bifidobacterium* spp., *B. breve*, *B. longum*, and *B. catenulatum* were significantly higher in the M-16 V group than in the controls. Additionally, colonization by M-16 V persisted for at least several weeks after the discontinuation of probiotics (Horigome et al. 2021). Li et al. (2004) suggested that early administration of *B. breve* had the beneficial effect of promoting the colonization of *Bifidobacterium* and reducing susceptibility to colonization by potential pathogens.

### Bifidobacterium lactis

In a previous cohort study by Chi et al. (2021), 138 PTIs were fed breast milk (BM, n = 31), probiotic formula (PF, n = 59) (*B. lactis*), or non-probiotic formula (NPF, n = 48) (Li et al. 2004), and the longitudinal variations in GM diversity and composition were explored. Diversity (Shannon index and Simpson indices) was highest in the PF group in the first week, and it was significantly higher than that in the BM group in the sixth week after birth. The NPF group had a greater relative abundance of *Enterococcus* (28.20%) than that of the BM (19.57%) and PF (9.57%) groups. *Bifidobacterium* was gradually enriched in all infants, with a larger proportion in the PF group than in the other two groups. The GM values of the three groups tended to be similar by week 12. Therefore, probiotic supplementation may affect GM colonization and reduce the number of some potential pathogens.

### B. longum subsp. infantis

A recent observational study (Nguyen et al. 2021) consisted of 77 PTIs indicated that *B. infantis* EVC001 reduced enteric inflammation. A higher abundance of *Bifidobacteriaceae*, with rapid development and significantly lower levels of key pro-inflammatory biomarkers, were detected in the *B. longum* subsp. *infantis* EVC001-treated group (n = 31) than in the control group (n = 46). Furthermore, *B. longum* subsp. *Infantis* EVC001 improved the functional capacity of the GM of PTIs for HMO utilization. In the ProPrems trial of very premature infants (n = 1099), supplementation with *B. longum* subsp. *infantis* BB-02, *Streptococcus thermophilus* TH-4, and *B. animalis* subsp. *lactis* BB-12 was associated with an increased abundance of *Bifidobacterium* soon after birth, resulting in a reduced NEC risk (Plummer et al. 2018). Commencing this supplementation within 5 days of birth was associated with an increased detection of probiotic species over the study period, suggesting improved subsequent colonization by probiotics (Plummer et al. 2021).

### Lactobacillus

#### L. reuteri

In a randomized placebo-controlled trial of 132 PTIs (< 1500 g) who received *L. reuteri* DSM 17,938 or a placebo from birth to the postnatal week, 86% of extremely LBW infants treated with *L. reuteri* were colonized with this probiotic strain during the neonatal period (Spreckels et al. 2021). A lower abundance of *Enterobacteriaceae* and *Staphylococcaceae* was observed in the *L. reuteri-*supplemented group during the first week. The composition and diversity of the GM differed between groups during the first month of life (Martí et al. 2021). At 2 years of age, no difference was found in the GM, and there was no effect on NEC or sepsis incidence (Spreckels et al. 2021; Martí et al. 2021). *L. reuteri* may be useful in improving feeding tolerance, promoting growth, facilitating defecation, and shortening hospital stays in PTIs (Cui et al. 2019; Wejryd et al. 2019).

### L. acidophilus

Greater *Bifidobacterium* (15.1%) and *Lactobacillus* (4.2%) abundances were observed in groups supplemented with *L. acidophilus* NCIMB701748 and *B. bifidum* ATCC15696 than observed in the control group (*Bifidobacterium* 4.0% and *Lactobacillus* 0%). *Bifidobacterium* abundance remained high after hospital discharge, suggesting successful long-term colonization, whereas that of *Lactobacillus* was reduced (Abdulkadir et al. 2016). Extremely LBW PTIs exposed to antibiotics supplemented with *L. acidophilus* and *B. longum* subsp. *infantis* had a higher relative abundance of *Bifidobacterium* and *Lactobacillus* than that of FTI controls. The FTIs had a higher abundance of *Streptococcus*, *Veillonella*, and *Haemophilus*. At 4 weeks and 4 months, the overall microbial diversity and resistome of the probiotic-supplemented infants were similar to those of the more mature infants. This indicates that probiotics may induce colonization resistance and alleviate the harmful effects of antibiotics on the GM and antibiotic resistome (Esaiassen et al. 2018).

Initiating probiotics shortly after birth has been recommended in several studies because of its benefits to the GM (Plummer et al. 2021), and the efficacy and safety of probiotics have been demonstrated. However, the effect of early probiotic supplementation is not sustained, as observed at 2 years of age. Additionally, the highly dynamic and individualized nature of the GM (Shao et al. 2019) limits studies examining the GM at an isolated time point, often resulting in the failure to identify differences between probiotic and placebo groups over time. Future research should consider the dosage, duration, and standard indications of probiotics, especially combined supplementation, evaluate the targeted and effective use of multiple probiotic strains to benefit the health status of the host, and explore whether there is a possible association between breastfeeding and the colonization efficacy of probiotics.

## Conclusions

The GM is strongly associated with the health status of PTIs. Although it is generally believed that GM colonization begins as the neonate leaves its mother, prenatal maternal status can pre-dispose the infant to dysbiosis before delivery. PTIs born via C-section or vaginally are initially colonized by different microbiota and exhibit different microbial distributions. Breastfed PTIs, or those supplemented with HMOs, may exhibit a more “normal” GM resembling that of FTIs. Antibiotic and probiotic administration may significantly influence the GM composition. The influence of all of the aforementioned factors subsides with age.

## Data Availability

Not applicable.

## Abbreviations

**C-section**: Cesarean section
**DHM**: Donor human milk
**FMT**: Fecal microbiota transplantation
**FTIs**: Full-term infants
**GA**: Gestation age
**GDM**: Gestational diabetes mellitus
**GM**: Gut microbiota
**HBM**: Human breast milk
**HMOs**: Human milk oligosaccharides
**IAP**: Intrapartum antibiotic prophylaxis
**LBW**: Low birth weight
**LOS**: Late-onset sepsis
**MOM**: Mother’s own milk
**NEC**: Necrotizing enterocolitis
**PMA**: Postmenstrual age
**PROM**: Premature rupture of the fetal membrane
**PTIs**: Preterm infants
**TLR**: Toll-like receptor
